# Leprosy and the Colonial Gaze: Comparing the Dutch West and East Indies, 1750–1950

**DOI:** 10.1093/shm/hkz079

**Published:** 2019-12-20

**Authors:** Stephen Snelders, Leo van Bergen, Frank Huisman

**Affiliations:** UMC Utrecht, PO Box 85500 HP Str. 6.131, 3508 GA Utrecht, The Netherlands

**Keywords:** leprosy, Dutch colonial empire, plantation economy, labour management, othering, compulsory segregation, colonial gaze

## Abstract

This article is looking at colonial governance with regard to leprosy, comparing two settings of the Dutch colonial empire: Suriname and the Dutch East Indies. Whereas segregation became formal policy in Suriname, leprosy sufferers were hardly ever segregated in the Dutch East Indies. We argue that the perceived needs to maintain a healthy labour force and to prevent contamination of white populations were the driving forces behind the difference in response to the disease. Wherever close contact between European planters and a non-European labour force existed together with conditions of forced servitude (either slavery or indentured labour), the Dutch response was to link leprosy to racial inferiority in order to legitimise compulsory segregation. This mainly happened in Suriname. We would like to suggest that forced labour, leprosy and compulsory segregation were connected through the ‘colonial gaze’, legitimising compulsory segregation of leprosy sufferers who had become useless to the plantation economy.

Leprosy has always triggered strong responses and policies, ranging from sympathy and philanthropy to disgust, exclusion and even compulsory segregation.^1^ The response to the disease is an important topic of studies of colonial medicine, with its specific focus on the relationship between rulers of European descent and a ruled population made up of non-white indigenous or imported people.^2^ While compulsory segregation of patients may have been the most visible response to leprosy, it was by no means universal. It may, in fact, be called the exception to the rule due to financial constraints and problems of distance and communication.^3^ In this article, we will argue that an important determinant factor in deciding on compulsory segregation was the way in which labour was organised in the colonies. Building on and paraphrasing Michel Foucault, who coined the concept of ‘medical gaze’ to refer to the power dynamics going on between doctor and patient in the clinic, we would like to introduce the concept of ‘colonial gaze’ to refer to the ways in which plantation owners, colonial administrators, doctors and surgeons legitimised compulsory segregation of leprosy sufferers who were no longer of use on the plantation or as labourers in general. Focusing on the case of the Dutch colonial empire, we will discuss the difference in response to leprosy in the Dutch West and East Indies from this perspective. We argue that leprosy only led to compulsory segregation in areas of direct Dutch rule and in colonies with plantation-intensive economic systems where large numbers of racial ‘Others’ were bound in slavery or involuntary servitude and in everyday contact with the colonisers.^4^

The colonial response to the disease has mainly been investigated in the context of late nineteenth– and twentieth-century imperialism.^5^ The argument in the literature runs as follows: by the end of the nineteenth century, leprosy had come to be seen as an ‘imperial danger’.^6^ Throughout the European empires, the spread of leprosy was presumed to be caused by the migration of non-white labourers who were supposed to endanger the health and lives of white people by introducing disease into the colony.^7^ Stigmatisation and compulsory segregation were used as repressive ‘tools of empire’, creating dichotomies between colonisers and colonised, centre and periphery, civilised and barbarous in an attempt to keep the danger at a distance.^8^

The case of the Dutch colonial empire, explored here, suggests that the relationship between colonial rule and leprosy cannot be generalised in this way. To begin with, segregation policies can be found well before the late nineteenth century. Secondly, colonial policies were closely connected to the interests of plantation owners and other entrepreneurs. Their interests often determined the response to leprosy and even influenced the framing of the disease itself. Rather than acting out of humanitarian inclination or political conviction, colonial elites in plantation economies were driven in their leprosy policies by economic interests and especially by the perceived need to maintain a healthy labour force and therefore to stop the spread of leprosy among the work force and to the white colonisers. This explains why compulsory segregation was introduced only in some parts of the Dutch colonial empire, remaining absent in others. Thus, while compulsory segregation as a general policy was rejected in the Dutch East Indies (now Indonesia), it was introduced very early on in Suriname (Dutch Guiana) in the West Indies (in 1790), remaining in force until the end of direct Dutch colonial rule (in 1950).^9^ In only two exceptional cases was compulsory segregation introduced in the East Indies. We argue that in all of these instances, a system of forced labour (either slavery or indentured labour) was in place. To be sure, the introduction of this system cannot be explained in a monocausal way, since there were important cultural, political and demographic differences between the East and the West Indies. Still, we argue that labour management—more in particular, the perceived needs in maintaining a healthy labour force and in preventing contamination of the white populations—contributed to the formulation of racial and sexual stereotypes of leprosy sufferers and to segregation policies in important ways. The perceived demands of the labour market in plantation colonies and the physical proximity of labourers led doctors and surgeons to creatively adapt classical humoral pathology to contemporary colonial needs. In doing so, they looked with what we call a ‘colonial gaze’. While in the ‘medical gaze’, as formulated by Foucault, the sufferer’s condition was separated from his or her bodily identity, in the colonial gaze, condition and (‘racial’) identity were intimately connected.^10^ As we will argue, this colonial gaze was instrumental in a process of ‘Othering’of the diseased bodies.

We will substantiate this claim by presenting case studies from the Dutch West Indies and the Dutch East Indies ranging from the seventeenth to the twentieth centuries. These case studies have been taken from research done by Stephen Snelders on Suriname and Leo van Bergen on the Dutch East Indies, using a wide variety of available unpublished and published primary sources. For Suriname, these include the archives of the colonial state in the Dutch National Archive in The Hague and the Surinamese National Archive in Paramaribo; the extensive reports of the colonial state to Dutch parliament; the archives of religious missions active in leprosy care (of the Roman Catholic Church of Suriname in Paramaribo, and of the Protestant missionary society held in the historical archive of Utrecht) and Surinamese journals and newspapers. In the case of the Dutch East Indies, besides of course again the National Archives in The Hague, the main archives used are those of the Mission Council and the Salvation Army in Utrecht and the Royal Netherlands Institute of Southeast Asian and Caribbean Studies (KITLV) in Leiden. Also, naturally, the National Indonesian Archives (ANRI) have been visited several times. Furthermore, information has been gathered from Dutch-Indian newspapers and from the *Medical Journal of the Dutch Indies* (GTNI), which was published from 1852 up until 1942.^11^

## The Framing and Management of Leprosy in Suriname

In 1667, the English ceded Suriname to the Dutch.^12^ The colony was brought under the control of a private company, the Society of Suriname, comprised of the Dutch West Indies Company (WIC), the city of Amsterdam and Cornelis van Aerssen van Sommelsdijck, a private investor who later became governor of Suriname. From that moment onwards, the Dutch created a thriving plantation economy based on African slave labour. By 1754, there were almost 1,500 Europeans and more than 33,000 slaves in Suriname. The Amerindians (the original inhabitants) and the Maroons (runaway African slaves) were living in the interior. They were autonomous and not integrated in the plantation economy. Thirty years later, the population had grown to over 2,000 Europeans and more than 50,000 slaves.^13^ By the 1780s, there were more than 400 plantations, cultivating and exporting sugar, coffee, cocoa, cotton and timber.

The transatlantic slave trade and the subsequent movement of millions of Africans across the Atlantic caused an epidemiological transition. As others have shown in more detail, forced migration of Africans to the New World transformed the disease environment in the Caribbean in profound ways. Yellow fever, filariasis, malaria and yaws were among the diseases that became rampant in the Caribbean, seriously threatening the success of European military operations and economic activities.^14^ The close proximity of European plantation owners to slaves of African descent prompted inquiries into the health and disease of the non-white population in the Caribbean more than in Asia.^15^ To European doctors and surgeons in the Caribbean, the African represented an important source of pollution.^16^ Especially in Suriname, leprosy came to be seen as a symbol of the African threat to Europeans.^17^ Leprosy served to remind the Dutch of the presumed importance of creating clear distinctions and upholding clear boundaries along ‘racial’ lines. The fear of leprosy led to compulsory segregation policies originating in Suriname *itself* rather than being imported from the metropolitan ‘centre’ of the colonial empire to its ‘periphery’.^18^ Segregation policies were developed from the perspective of a ‘slaveholder’s knowledge’—with ‘slaveholders’ not just referring to actual owners of slaves but also to ‘many more with a direct or indirect interest in slaveholding through family connections or professional and business arrangements’.^19^

This process started in the 1750s, when an unknown disease among slaves was observed, the signs of which reminded doctors of descriptions of leprosy in medical textbooks.^20^ It was called ‘boasie’, after the name of the place in Africa where the disease was believed to originate.^21^ Contemporaries routinely equated boasie with *elephantiasis graecorum* or *lepra arabum*, i.e. leprosy, the dreaded disease of the Middle Ages.^22^ In the eighteenth century, many European doctors and laymen regarded the disease as highly contagious.^23^ In Suriname, healthy persons were advised to stay away from sufferers, not to enter their dwellings, not to touch them nor even breathe the same air.^24^ To many slaveholders, leprosy seemed to endanger the health of their slave labour force and with it the proper functioning of the Surinamese economy. Profits would be seriously at risk if changes in the disease environment were not met by medical intervention. The colonial framing of leprosy that followed decisively influenced dealings with leprosy in Suriname.

Over the course of the eighteenth century, leprosy became increasingly visible in public life. It was increasingly perceived as a threat calling for a response. Whereas a 1728 edict of the colonial government—prohibiting slaves with potentially contagious diseases from traveling on public roads—did not yet mention leprosy, an edict of 30 years later did. It is an indication of the increased visibility of leprosy in public life during the intermediate years.^25^ The 1761 edict was ineffective, however, as slave owners continued to send afflicted slaves to surgeon’s shops in Paramaribo. Therefore, a new edict was issued in 1764, prohibiting the treatment of slaves in the city. Henceforth, surgeons were expected to travel to the plantations to attend to the slaves.^26^ In 1780, the governor of Suriname issued another edict explicitly prohibiting the sale of leprous slaves, a measure he would never have taken had this not been common practice.^27^ In 1790, the colonial government issued even stricter measures, introducing compulsory segregation that forced slave owners to report possible leprosy cases among their slaves to the *Collegium Medicum*. When this board of medical supervisors diagnosed a slave as being affected by leprosy, the affected slave would have to be moved to a special leprosy colony called ‘Prevention’(*Voorzorg*).^28^

Leprosy was not the only contagious disease causing concern for the colonial government. Yellow fever, small pox, all kinds of other ‘fevers’and ‘poxes’, as well as the skin disease yaws all seemed to have been introduced to Suriname through slave ships and other vessels. These diseases became the object of hygienic policies of the colonial government. Although some of the diseases posed more of a threat than leprosy, none of them received the moral and sexual connotations of leprosy.^29^ The edict of 1790 can be considered as a codification of this colonial framing of leprosy, which had materialised during the preceding decades. The German slave doctor Godfried Wilhelm Schilling had played an all-important role in this process.^30^ As one of the few physicians working in the colony of Suriname, and one who conducted medical examinations of newly arrived slaves, Schilling supplied the scientific ‘evidence’ and underpinnings of the repressive policy of isolation and segregation based on an humoral understanding of disease. He claimed that leprosy was caused by a ‘special substance’, ‘a certain poison’ that was brought by slaves from Africa to the Americas and that could become virulent when climate or inadequate diet had weakened a person’s constitution. Abnormal thickening of bodily fluids prevented their healthy evaporation. If the weakened body was then contaminated by the leprosy poison, it contracted the disease. The poison itself spread through physical contact: either by sexual intercourse or by contact with the exudations of ulcers and wounds.^31^ One could stop its progress only by adopting a healthy diet and lifestyle.

Schilling went on to ‘racialise’ the traditional humoral understanding of the disease. Race was an essential category to him in understanding the aetiology and epidemiology of leprosy and important in determining the measures that needed to be taken. He emphatically pointed out that Africans were the carriers of the disease. In doing so, he justified the edicts aiming to isolate leprosy sufferers. Schilling felt that whenever Europeans had physical contact with Africans, they were in danger of being contaminated. There were distinct moral connotations to this view: Africans were considered prone to contract the disease because they lacked the self-control and level of civilisation needed to withstand the leprosy poison. At first glance it seems that Schilling put the blame entirely on the Africans. However, European males were in danger of contracting leprosy as well, in particular when they had sexual intercourse with slaves. The moment they lost their self-control, stooping to the level of a race that was held to be inferior, they were prone to contract leprosy as well. The more one was ruled by lust, the bigger the risk of leprosy spreading from slave to master.^32^ Thus, moral, social and medical degeneration went hand in hand, endangering European dominance in a society largely consisting of Africans.

Schilling framed leprosy as a disease of African origin, tainted with negative racial and sexual connotations, caused by unhealthy living conditions and related to low sexual standards. In his colonial gaze, the condition of leprosy was intimately connected to the identity of the African sufferer, both bodily and psychologically. His framing of leprosy was fully in line with the general views of the Dutch in Suriname.^33^ They had come to see leprosy as a problem of geography and race, as a threat to the functioning of the slave economy and ultimately as a danger to European dominance.

### A Policy of ‘Great Confinement’, 1815–1863

During the decades preceding 1863 (the year of the abolishment of slavery), the treatment of leprosy sufferers in Suriname came close to a Great Confinement. This concept was famously coined by Michel Foucault in his *Madness and Civilization* to designate the confinement of madmen in seventeenth-century Paris in ‘enormous houses’. These houses, Foucault claimed, were not medical establishments, but rather ‘an instance of order, of the monarchical and bourgeois order being organised in France during this period (…) More than one out of every hundred inhabitants of the city of Paris found themselves confined there’.^34^ In Suriname, close to one out of every hundred inhabitants found themselves condemned or suspected of having leprosy. After medical examination, those who were diagnosed with leprosy were confined to the leprosy asylum of Batavia (not to be confused with the VOC capital of the same name in the Dutch East Indies), especially when they were slaves or poor. In case they were members of wealthy families, they were segregated in their own homes.^35^ Batavia was opened after *Voorzorg* was closed. The leprosy asylum was not primarily a medical establishment: sufferers were isolated in a place far away in the jungle, with hardly any medical attendance. Batavia’s function was to maintain colonial order, not to care for or to treat patients.

Doctors in early nineteenth-century Suriname routinely embraced Schilling’s sexualised and racialised discourse on leprosy. They complained about the supposed fatalism of Africans, slaves and leprosy sufferers and expressed fear for a ‘return’ of the disease to Europe. Exemplary in this respect is a treatise on leprosy by the Dutch surgeon Andries van Hasselaar. It was published in 1835, after Van Hasselaar had served 12 years in Suriname. In his *Description of elephantiasis and leprosy, prevalent in the colony Suriname*, Van Hasselaar presented the slaves as lazy, their lifestyle as slovenly, their personal hygiene as insufficient and their diet as unhealthy. Last but not least, African slaves believed in a so-called *treef*: a taboo animal that everyone was believed to receive at birth and that could not be eaten—or used otherwise—without the direst consequences, including the contraction of leprosy.^36^ ‘The [belief in] predestination and its resulting carelessness to protect oneself against contagion, bring many to [contribute to] the expansion of the contagion’, Van Hasselaar wrote.^37^ When someone was predestined to contract leprosy, it was useless to take measures against it in the Afro-Surinamese view. To Van Hasselaar, the Afro-Surinamese were keeping the threat of contagion alive because of their fatalism and laziness. He also adopted Schilling’s sexualisation of the disease. According to Van Hasselaar, one of the results of the spread of leprosy in Suriname was the presence of a group of highly sexually charged female sufferers at an early stage of the disease. Because of the animal urges of European men, this threatened to transmit the disease to Europeans and even to Europe. For this reason, Van Hasselaar advocated a medical examination of all Europeans returning to the Netherlands, with a special focus on leprosy. His advice is proof of the deep-seated fear of a ‘return’of leprosy to Europe.^38^

In 1828, a major change in Dutch slave legislation necessitated new leprosy policies. Before that year, slaves had the legal status of mere objects; they could not own possessions or be married. In 1828, their legal status was changed from object to person, making new legislation on leprosy necessary. For the next 100 years, the leprosy edict of 1830 was to serve as the legal framework for leprosy policies. It was built on the foundations of the edict of 1790 that had first formulated the principle of compulsory segregation. Extensive powers of criminal investigation to track down suspected sufferers were given to the police of Paramaribo, the capital of the colony and the only settlement with a significant population outside of the plantations. The police were supposed to make a general visitation of all dwellings in Paramaribo once every 3 years, starting in 1831. Before they were put up for auction, slaves were to be examined by the city physician. Rewards were promised for every leprous slave that was brought before a medical Committee of Investigation (*Commissie van Onderzoek*). The Committee directed its ‘colonial gaze’ especially (although not exclusively) at slaves, brought before them by their owners. When in doubt about the diagnosis, the Committee declared the examined to be ‘suspect’. In case this concerned a slave, he would have to carry a ‘suspect’-sign around his neck.^39^ Suspects were brought to Boniface, a special government terrain near Paramaribo, where they were re-examined after 1 year. Depending on the diagnosis, they were then either transported to Batavia to be isolated in the jungle or returned to their owners.^40^ In the early 1830s, the search for the infected had become so intense that it endangered the slave market. Already in June 1831, a new edict toned down the strictest measures of the edict of the previous year, because they could lead to ‘discomfort among good residents’.^41^ In general, it remains the question whether all measures were strictly carried out. Even so, between 1831 and 1859, 1,344 persons were declared infected—being 46 per cent of all examined persons. The impact of the Great Confinement policy becomes clear when we compare the number of infected people with total population figures. According to one estimate, the population in 1837 numbered 50,467 slaves and 8,495 freemen.^42^ Between 1831 and 1837, the Committee examined 952 people, declaring 434 of them infected. The total number of examinations must have been even higher, since examinations were carried out on the slave markets as well. Since almost all people who were examined or diagnosed as infected were slaves, almost 2 per cent of the total slave population in 1837 had been confronted with the ‘colonial gaze’ on leprosy, something that did not occur in the case of any other disease.

After 1849, the population of Batavia dwindled. This did not mean that segregation policies had been revoked or modified—quite the contrary. In 1853, and again in 1855, leprosy edicts were issued, which announced stricter measures than before. Henceforth, there were to be school investigations on a yearly basis, infected persons had to be reported within 24 hours, a general police visitation of all dwellings was left to the discretion of the police commissioner and a health certificate was mandated for every request for manumission (release) of a slave.^43^ In spite of all this, the number of sufferers incarcerated in Batavia decreased. How is this to be explained?

One could hypothesise that this downward trend was related to increasing medical doubts about the contagious nature of leprosy, as triggered by *Om Spedalskhed* (1848), a book written by the two Norwegian leprologists Boeck and Danielsse, who gave a primary role to heredity in the aetiology of the disease. Their book had become accessible to the international medical community through a French translation. Less than 20 years later, in 1867, the British Royal College of Physicians published a report in which leprosy was constructed as a hereditary disease, denying the role of contagion.^45^ Not all medical professionals subscribed to this paradigm shift, but in Suriname, the shift did not happen at all—quite the contrary. Different views on the aetiology of leprosy were discussed in Dutch medical journals and defended in MD theses. While in general contagion was questioned in the Netherlands and in the East Indies, Suriname kept to its own views.^46^ Clear proof of the continued belief in the contagious nature of leprosy was the MD thesis of Charles Drognat Landré, the son of a Dutch physician in Suriname. He defended it at Utrecht University in 1867, almost 100 years after Schilling had defended *his* thesis at the same university. Contrary to some of his colleagues in the Netherlands, Drognat Landré took a clear stance against the hereditarian view in *The contagiousness of leprosy*. On the basis of his reading of the history of leprosy in Suriname, he maintained that the disease was transmitted through physical contact between humans.^47^ The ‘leprosy poison’ of Schilling transformed into the ‘contagium’of Drognat Landré, who explicitly referred to the work of his predecessor.^48^ Drognat Landré opposed the views of Danielssen and Boeck by claiming ‘without contagium, no leprosy’.^49^

Quite a different factor might explain the decline of the confinement of leprosy sufferers in Suriname: the impending end of slavery. In 1853, a committee was installed in the Netherlands to prepare the abolition of slavery. Although it was to take another 10 years before the plans materialised, the intention to abolish slavery really set things in motion. We would like to argue that the steep decline in the number of leprosy sufferers in Batavia was caused by monetary considerations. Plantation and other slave owners had always been vital in bringing suspected slaves before the Committee of Investigation. Emancipation would give them financial compensation for their released slaves, but not for all of them. In 1857, an early proposal of a Dutch government committee for the Emancipation act specifically excluded slaves with leprosy or elephantiasis from compensation to the owners.^50^ This exclusion was also the final word. A committee of three physicians appointed by the Governor was to examine all slaves suspected of having leprosy or elephantiasis. If these slaves had not been declared healthy 1 year after the examination, there would be no financial compensation.^51^ We therefore suggest that the major reason for the decline in the Great Confinement policy since the mid-1850s was the growing awareness of impending Emancipation: slave owners were unwilling to report leprosy among their slaves because this implied losing financial compensation when Emancipation would take effect.

### The Modern Colonial State and Compulsory Segregation after 1863

**Fig. 1.**
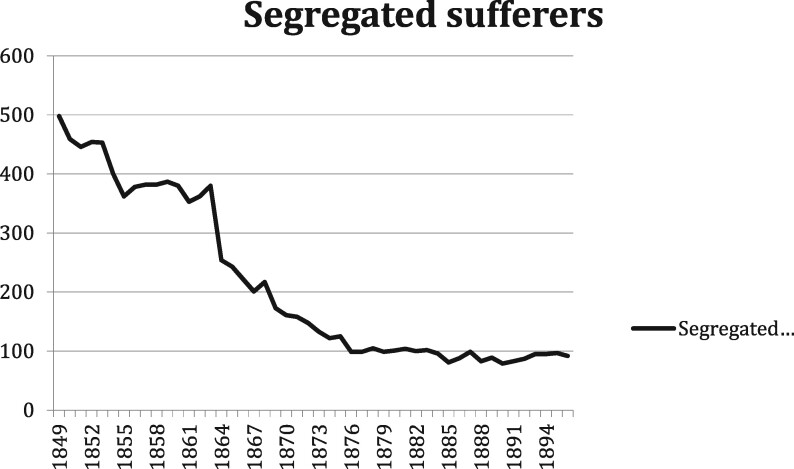
Segregated sufferers in Batavia, 1849–1897.^44^

**Fig. 2.**
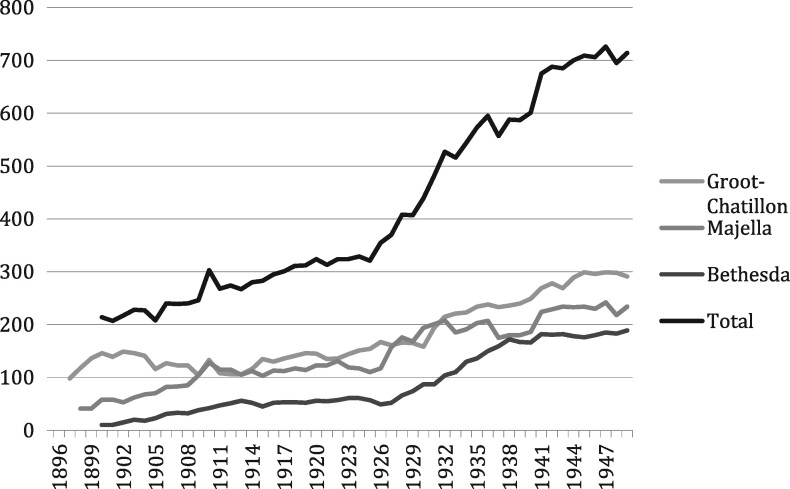
Numbers of patients in the asylums, 1896–1949^55^. Groot-Chatillon = government asylum, Majella = Catholic asylum, Bethesda = Protestant asylum. NB: on closure of Batavia, its remaining sufferers were transferred to Groot-Chatillon.

After slavery was abolished in 1863, Surinamese society was transformed by the immigration of Asian indentured labourers—mostly coming from British India and Java—taking the place of former slaves on the plantations and becoming an essential part of the Surinamese labour force. In the twentieth century, large-scale agriculture would lose its economic importance to mining (products included bauxite, gold and oil). Gradually, a modern colonial state developed. Reforms were put in place aiming at the assimilation of former slaves and their descendants, who were now considered to be Dutch citizens living in overseas Dutch territory.^52^ The end of slavery did not, however, mean the end of compulsory segregation in Suriname. The social and cultural heritage of slavery continued to have its impact on dealings with leprosy. Post-Emancipation Suriname remained permeated by what has been called a *culture of domination*. The colonial state, social and religious organisations and employers attempted to exercise authoritarian control.^53^ At the same time, fear of leprosy sufferers remained. By 1930, 80 per cent of those diagnosed with leprosy were still Afro-Surinamese.^54^ After the number of segregated sufferers in Batavia had declined (Figure 1), they rose again after three new modernised asylums had been opened in the 1890s to replace Batavia (Figure 2).

Until well into the twentieth century, leprosy policies developed in the slave era continued to have their impact. Although a new leprosy edict issued in 1929 formalised a more ‘humane’ medicalised regime in the asylums, policies of surveillance, detection and compulsory segregation remained in place.^56^ During the final decades of direct Dutch colonial rule in Suriname, leprosy policies were still characterised by the general ‘culture of domination’. Thus, leprosy policies in the twentieth century were Janus-faced. On the one hand, they were based on the latest medical views on leprosy: sufferers should be treated as patients, not criminals; medical treatment should be encouraged and life in the asylums should be humane. On the other hand, leprosy remained associated with race and labour and with descendants of Afro-Surinamese slaves. Sufferers with a non-European background continued to be framed as being unwilling or unable to cooperate.^57^ In other words, the colonial gaze of European doctors and administrators continued to make an interpretation of the bodily condition of the sufferers related to their perceived ‘racial’ identities. Only when effective medication became available in the 1950s was compulsory segregation abandoned in Suriname.

## The Framing and Management of Leprosy in the Dutch East Indies

The Dutch East Indies have always been much more heterogeneous than Suriname. The archipelago consisted of a great variety of ethnicities, cultures, languages and societies that to a large extent remained autonomous. Given the complex power structures and the sheer size of the Dutch East Indies, it was difficult for the Dutch to gain control. Also—and unlike the situation in Suriname—the emphasis of the policies of the Dutch East Indies Company (VOC) was on trading rather than on the production of agricultural products. As a consequence, Dutch territorial rule was much more limited than in Suriname. Outside of Java (the most important island) and the Moluccas, the Dutch had very little power in the Archipelago until the nineteenth century. But even on Java, the Dutch behaved as a ‘reluctant imperialist’, mainly concerned with maintaining their influence on trade and keeping the balance of power between the different Javanese principalities.^58^ Whereas compulsory segregation became the rule in Suriname, it was the exception to the rule in the Dutch East Indies.

One of these exceptions was Batavia on Java. This city was the centre of Dutch power in the archipelago of the East Indies and has been called a ‘Dutch town in the tropics’.^59^ Together with the Moluccas, it was one of the few places in the archipelago where the Dutch were living in close contact with other parts of the population. Most of the people living in Batavia were not of European descent. By 1700, approximately 13,000 out of 20,000 of those living within the town walls were slaves. Of a further estimated 100,000 people living outside the walls, 30,000 were slaves. Originally these slaves were imported from India, later on from other islands in the archipelago, with a minority being imported from the east coast of Africa. Many of them worked as domestic servants. During the first half of the seventeenth century, their labour was essential in the construction and maintenance of the fortifications of Batavia, which was surrounded by Javanese who at times were hostile. By the eighteenth century, when the number of slaves had increased significantly, many of them were employed in industries such as sugar refineries and liquor distilleries. Their masters were almost as often European as they were Chinese, Arab, Indian or Javanese.^60^

As in Suriname, the European rulers of Batavia constituted only a small minority of its population. Both within and outside the town walls of Batavia, the Chinese had a very significant presence. They were active as artisans, merchants, fishermen, horticulturalists or plantation coolies. For this reason Batavia has also been called a Chinese colonial town under Dutch protection—at least until 1740, when Europeans rioted against the Chinese presence and murdered a significant number of their Chinese neighbours.^61^ The 5,000 servants of the VOC were living in Batavia on a temporary basis, a large part of them staying on Company ships in the harbour. On top of that, there were around 1,500 Europeans and 700 Mestizos in Batavia.^62^ Formally Batavia was a segregated town, with ethnic groups living in separate neighbourhoods, each with their own customs and economic activities. In reality, however, different ethnic and cultural groups mingled on an everyday basis, and inter-racial marriage occurred frequently. Whereas in the West Indies, the boundaries between Europe and Africa were strictly guarded, in the East Indies, it was difficult to tell where Europe ended and Asia began.^63^

The first time that the isolation of (three) leprosy sufferers is mentioned is around 1655.^64^ From then onwards, preoccupation with leprosy increased. A 1667 decree regulated the admittance and treatment of sufferers of the ‘horrendous and contagious disease called leprosy (*laserye*)’ in a leprosy home built just outside the city walls of Batavia.^65^ It was soon considered to be too close to town, so a new place for the leprosy asylum was established in 1679 on the island of Purmerend, thus guaranteeing better isolation of sufferers. An edict of 1681 decreed the compulsory segregation of sufferers.^66^ Suspected sufferers were to be examined by a committee of physicians and surgeons.^67^ One year later, district wardens were ordered to report suspected sufferers to the Governor-General.^68^ The 1682 edict was specifically intended for ‘common people’ (*gemeene luijden*), while wealthy patients were allowed to isolate themselves outside of town on the condition that they did not mingle with other people. They could even return to Europe, until this was prohibited by the VOC in 1695.^69^

Initially it had been unclear if leprosy was indigenous or had been brought to Java from elsewhere. Out of seventeen people diagnosed with the disease in 1682, six were (judging from their surnames, which is not conclusive) Dutch or Indo-European, while the others came from China, Thailand, India (Malabar), Malaysia (Malacca) and Indonesian islands such as Bali, Banda and Timor. These were all territories where the VOC had an active presence as trading company. All sufferers were sent to Purmerend, where Willem ten Rhijne was one of the two governors. Being a physician, he was expected to diagnose people suspected of suffering from leprosy.^70^ In 1687, he published a treatise on leprosy in which he framed the disease much like Schilling would do more than 80 years later. There was, however, a very significant difference between the two. Both were immersed in Galenic humoral medicine. Like Schilling, Ten Rhijne believed that for leprosy to become manifest, the transmission of a certain unspecified poison was necessary—either by physical contact or through the air. For the disease to develop, the constitution needed to be weakened—either by climate, an unhealthy lifestyle or sexual contact. Like Schilling, Ten Rhijne believed in the increased sexual appetite of leprosy sufferers, which would deteriorate their condition even more.^71^ An important difference between the two was that Ten Rhijne did not racialise the disease. Although he did worry about the spread of the disease as a result of the slave trade,^72^ he did not frame leprosy as the disease of an inferior ‘Other’, like Schilling did. The ‘colonial gaze’ so paramount in Suriname is not evidently present in Ten Rhijne.

Although compulsory segregation was introduced in Batavia, the history of the Purmerend asylum shows that leprosy never became the scare that it was in Suriname, perhaps because of its lesser incidence. The asylum was not limited to leprosy sufferers: sufferers of ‘Venus disease’ (syphilis) were taken there as well, as were all others whose looks were deemed harmful for pregnant women and the community at large.^73^ Over the course of the eighteenth century, the total number of patients—the majority of them slaves—dwindled.^74^ Fearing an English bombardment in 1795, the last leprosy sufferers living on Purmerend (eleven in total) were moved to the town of Batavia, after which the asylum was effectively closed.^75^

Like the asylum Voorzorg in Suriname, Purmerend had primarily been an asylum for slaves, reflecting the character of the town of Batavia as a slaveholder’s society. Batavia’s socio-economic situation, its large share of forced servitude and the close physical contact between Europeans and non-Europeans all contributed to making the town one of the very few places in the East Indies where the Dutch introduced compulsory segregation.

### Rejection of Compulsory Segregation

As a counterpoint to developments in Suriname, we will discuss the choices made by Dutch colonial government in the East. In 1865, it made the hereditarian view on the aetiology of leprosy the official cornerstone of its policies. This choice reflects the vastly different context of colonial rule in the East Indies as opposed to Suriname.

In the nineteenth century, a system of indirect rule was introduced in the East Indies, in which Javanese communities produced coffee, tea and sugar for the Dutch. The resulting political structure can be characterised as an association of Dutch and Javanese elites. Elsewhere in the Archipelago, Dutch political power remained mostly nominal and in fact non-existent. Only by the end of the nineteenth century did the modern colonial state start to extend its sovereignty to the other islands with military and other means.^76^ Distance between the Dutch and their subjects was therefore in general larger in the East Indies than in Suriname. As a consequence, in the Dutch East Indies, leprosy was never considered the danger it was perceived to be in Suriname. Medical services, even in a relatively ‘European’ town like Batavia, were focused on the military, leaving little personnel for civil health services.^77^ Even in Batavia, public health was not considered a major concern of government, and attempts to improve living conditions for non-Europeans were virtually non-existent.^78^

Still, as in Suriname, Europeans in the East Indies were concerned about the effects the climate might have on their health. For many Europeans in the Dutch East Indies, a non-specific but pervasive sense of danger was always present.^79^ However, these fears were focused on cholera and other diseases rather than on leprosy, which was a disease with a relatively minor occurrence. Moreover, since the plantations on Java were not controlled directly by the Dutch state and sufferers did not travel to towns with a large Dutch presence, anxiety about leprosy never rose to the levels of Suriname. In territories controlled directly by the Dutch, leprosy sufferers were isolated because they travelled the roads as beggars—not because they showed signs of leprosy. When in 1816, after the Napoleonic wars, the newly created kingdom of the Netherlands retook possession of the Dutch East Indies, beggars’ hospices were established, especially on Java. They arose in towns like Batavia, Surabaya, Tegal and Semarang, their main aim being to keep beggars with disturbing deformations out of sight. It can safely be assumed that many poor leprosy sufferers ended up in one of these beggar’s hospices.^80^ Partly because of the limited power of the Dutch, partly because of the system of indirect rule, there was no general leprosy policy in place.^81^

Only a minority of the Dutch colonial administrators^82^ on Java advocated complete segregation. The others either chose to leave policy to the discretion of the local Javanese authorities—which was in line with the principle of indirect rule—or rejected the idea of segregation altogether.^83^ In Javanese conceptualisations, leprosy, like other diseases, could be caused by worms, poison, unhealthy winds, magic or spirits punishing transgressions against customs and laws, rather than by contagion.^84^

By 1865, the Dutch opponents of segregation had won the debate. In that year, and in complete contradiction to policies ruling Suriname, the colonial government of the Dutch East Indies declared leprosy to be a non-contagious disease. Sufferers—either indigenous or European—could therefore not be forced into segregation.^85^ An edict of 1870 proclaimed that the colonial government would not finance the admission of new leprosy sufferers in the asylums.^86^ Together, these edicts suggest that financial considerations played a considerable role in these rulings, as did the sheer size of the Archipelago.^87^ Building and maintaining leprosy asylums was not only too costly but considered to be a waste of money as well. Contrary to Surinamese slave society, contacts between Europeans and indigenous leprosy sufferers were simply too limited to trigger a ‘colonial gaze’ and lead to strict segregation policies. Whereas the Dutch West Indies clung to contagionism to serve the interests of their plantation economy, the Dutch East Indies embraced hereditarianism. In the Dutch East Indies, the threat of leprosy was too diffuse and the costs of building leprosy asylums too high to merit segregation policies. Thus, it may be argued that the dominant hereditarianism of the day offered a convenient rationale for a policy of non-intervention in the Dutch East Indies. It is significant that while in Suriname the number of sufferers was meticulously counted, similar statistics are lacking for the Dutch East Indies. Estimations made after 1900—running into tens of thousands, even 150,000 sufferers^88^—had emotional rather than real value. They were an indication of a rather abstract popular fear in the absence of concrete leprosy policies.

In 1897, an international conference on leprosy was held in Berlin. Acknowledging the findings of Gerhard Armauer Hansen, who had identified the leprosy bacillus in 1873, leprosy was declared a contagious disease. The Dutch delegation in Berlin consisted only of representatives coming from the Dutch East Indies; there were none from Suriname.^89^ This is remarkable for at least two reasons: first, because Surinamese physicians had much more experience with leprosy, and secondly, because they had continued to adhere to the contagion theory. The choice seems to suggest that in choosing representatives for the Berlin conference, the economic relevance of a colony was considered to be of more importance than the medical expertise of its physicians. By the end of the nineteenth century, the East Indies were much more important to the treasury of the Netherlands than the West Indies. However this may be, after the Berlin conference, leprosy policies in the Dutch East Indies did not change much. Despite international fears about the transmission of the leprosy bacillus due to global migratory movements, the number of leprosy patients in the actual care of Dutch doctors remained almost negligible.^90^

While the Berlin conference did not make much of a difference to leprosy policies, what did cause anxiety was the arrival of Chinese labourers in the Dutch East Indies in the 1890s.^91^ In the public media, the Chinese—who had traditionally been viewed with suspicion by the Dutch—were held responsible for an increase in the transmission of leprosy.^92^ The arrival of Chinese labour migrants stimulated constructions of their ‘Otherness’, and leprosy became associated with the Chinese and their supposed racial inferiority, as happened in other European colonies.^93^ Still, even the influx of Chinese did not lead to a change in general policies, let alone to compulsory segregation. Measures were limited to the revoking of the government edict of 1865 in 1908 and to prohibiting leprous children from attending school.^94^ Unlike the situation in Suriname, the physical distance between colonial rulers and ruled was too large to invest in large-scale measures. There was, however, one notable exception to this rule: the Deli tobacco plantations on the island of Sumatra, a place where labour conditions were very similar to those on the slave plantations in Suriname. This, in turn, led to a very similar response to leprosy.

### Compulsory Segregation as Labour Management in Deli, Sumatra

For a long time, Dutch presence on Sumatra had been virtually non-existent. In 1866, a 30-year old adventurer from Amsterdam named Jacob Nienhuys received the lease of 30,000 acres of land from the ruler of eastern Sumatra, the Sultan of Deli. Keen on cultivating tobacco, Nienhuys co-founded the Deli Company (*Deli Maatschappij*) that would ultimately cultivate as much as 300,000 acres. By 1876, there were 40 plantations in a territory of which Medan was the centre. Since the Malayans on the coast of Sumatra and the Bataks in the interior were not interested in working these plantations, the Deli planters needed to import labourers from elsewhere. The Deli Company decided to hire Chinese and, later on, Javanese migrant labourers. In 1881, there were 15,000 Chinese and 2,000 Javanese working on the plantations. By 1900, their numbers had grown to 55,000 Chinese and 34,500 Javanese. Labour conditions were appalling and very similar to those on Caribbean slave plantations. The Dutch coolie ordinance for the east coast of Sumatra in 1880 granted planters complete control over their labour force.^95^ Slavery and forced servitude are apt qualifications to describe the physical and social violence implied in Deli labour relations.^96^

The patterns of response on plantations in the East and the West were quite similar: although Chinese migrant labourers were needed to work the plantations and make them profitable, the Dutch viewed them as ‘invading’ their world. Like the slaves in the West Indies, the coolies were seen as racially ‘Other’and inferior. The ‘dog eaters’ and ‘(pig)tail bearers’—as the Chinese were called—were perceived as the Yellow Peril.^97^ By 1910, there were pleas for an immigration policy examining labourers, checking them for signs of leprosy.^98^ The Javanese, in their turn, had for centuries been constructed by the Dutch as racially inferior and passive, lacking the vitalism of the white race and awaiting the civilising mission of the Dutch.^99^

As in Suriname, this process of ‘Othering’ was of central importance in developing a ‘colonial gaze’ in looking at leprosy sufferers and stimulating leprosy segregation policies. In 1890, the Deli Company decided to open a small asylum in Medan, which was able to hold 35 patients. Officially, admission was on a voluntary basis, but reality was different.^100^ Individuals who had been expelled from plantations and were living as vagrants were forcibly admitted to the asylum. Very soon, the asylum proved to be too small, and in 1892, its capacity was increased to 100. Most of those admitted were Chinese: in 1903, there were 78 Chinese on a total of 83 patients. Living conditions were very bad, and leprosy sufferers living in the asylum were ‘infused with a hatred of the world’, according to one Dutch physician working for the Deli Company. Lawlessness was the norm in the Medan asylum. Theft was common, and in 1904, one sufferer was even killed by a fellow patient. Between 1890 and 1903, a total of 419 patients were admitted, 147 of whom escaped. Patients were transferred from the government prison to the asylum, in order not to ‘contaminate’ the prison.^101^ Among the patients were those admitted for reasons of prevention, those who were sentenced by the police and those who did forced labour.^102^ Without irony or sarcasm, the Public Health Service referred to the asylum in Medan as the ‘leprosy sufferers’ prison’ (*leprozengevangenis*). In 1907, the plantation owners in Deli founded an association for building and maintaining a new leprosy asylum that was to be organised on a compulsory basis and directed at the segregation of ‘poor mendicant sufferers’ (*armlastige vagebondeerende leprozen*).^103^ The new asylum in Deli, on the island Pulu Sitjanang, was opened in 1914. An edict of the previous year made it possible to send leprous vagrants with ‘hideous’ signs of the disease to the asylum.^104^ The diagnosis ‘hideous’ was very flexible, and Dutch colonial doctors on Sumatra’s east coast considered admission of people with *any* sign of leprosy advisable.^105^ It should not come as a surprise that the majority of the Pulu Sitjanang population was Chinese: first, because the majority of the plantation workers was Chinese and secondly, because all Chinese immigrants were suspected to be infected with leprosy.^106^ By 1931, the asylum had developed into a ‘dumping ground’ for more than 400 people, while there was space for only 240.^107^

It is remarkable that all of this happened while Dutch colonial government still officially rejected compulsory segregation. But it is easier to understand when we realise that building a leprosy asylum and putting compulsory segregation policies in place was not a decision taken by the colonial state in the context of public health. Rather, it was a private initiative taken by the planters of Deli who—as the plantation owners of Suriname had been—were driven by economic interests and the perceived needs of labour management. These plantation owners virtually controlled the government in the region and could therefore direct a colonial gaze and execute segregation policies in order to maintain a healthy labour force.

## Conclusion

This article has emphasised the dimension of forced labour—both slavery and indentured labour—in the field of leprosy history. By comparing leprosy policies in two very different colonies belonging to the same colonial empire, we have tried to show how responses to leprosy were driven by the specific *local* interests of plantation owners, company officials and colonial governments. The existence of forced servitude by non-Europeans under the direct rule of Europeans turned out to be an important variable in the development of compulsory segregation policies. The economic interests of planters and the perceived needs of labour management were driving forces in shaping the response to leprosy. Wherever close contact between European planters and a non-European labour force co-existed with conditions of forced servitude, the Dutch response was to link leprosy to racial inferiority in order to legitimise compulsory segregation. Wherever they were in close everyday contact with slaves, the Dutch felt the need to create social and psychological distance. This is what happened in the plantation colony of Suriname. In the Dutch East Indies however, there was hardly a need for the management of labour by the Dutch. There were only two exceptions to this rule: one concerned early-modern Batavia on Java (now Jakarta), the other Deli on Sumatra at the end of the nineteenth century. Both cases suggest that forced servitude by non-Europeans under the direct rule of (and therefore in direct contact with) Europeans was an important variable in the creation of a regime of compulsory segregation of leprosy sufferers.

Thus, under specific circumstances, colonialism led to the exclusion of the leprosy sufferer as the ultimate Other. In this process, socio-economic factors have been of fundamental importance in the Dutch colonial empire. In these cases, colonial medicine was more than simply a tool of empire. It was pivotal in incorporating the dimension of labour in a colonial gaze that intimately connected the condition of leprosy with the subjective and bodily identity of the sufferers, thus directing, as well as serving the colonial project. We hope that our discussion of the relationship between leprosy and forced labour—subsumed by the concept of the ‘colonial gaze’—may prove helpful in understanding the fate of leprosy sufferers in other colonial contexts as well. 

## Footnotes

1 Rod Edmond, *Leprosy and Empire: a Medical and Cultural History* (Cambridge: Cambridge University Press, 2006), 1. In order to avoid anachronism, we decided to use the word ‘leprosy’ rather than ‘Hansen’s disease’. To ‘reduce’ leprosy to an infectious disease would distract from its moral and socio-political connotations. This would have been counter-productive for our project, which seeks to understand colonial public health governance by highlighting these dimensions.

2 On leprosy in colonial societies, see Wolfgang Eckart, *Medizin und Kolonialimperialismus. Deutschland 1884–1945* (Paderborn: Schöningh, 1997); Jane Buckingham, *Leprosy in Colonial South India: Medicine and Confinement* (Houndmills: Palgrave, 2002); Alison Bashford, *Imperial Hygiene: a Critical History of Colonialism, Nationalism and Public Health* (Houndmills: Palgrave Macmillan, 2004), esp. chapter 4; Edmond, *Leprosy and Empire*; Warwick Anderson, *Colonial Pathologies: American Tropical Medicine, Race, and Hygiene in the Philippines* (Durham: Duke University Press, 2006), esp. chapter 6; Michelle T. Moran, *Colonizing Leprosy: Imperialism and the Politics of Public Health in the United States* (Chapel Hill: University of North Carolina Press, 2007); Ki Che Leung, *Leprosy in China: A History* (New York: Colombia University Press, 2008); Kah S. Lo, *Making and Unmaking the Asylum: Leprosy and Modernity in Singapore and Malaysia* (Petaling Jaya: Strategic Information and Research Development Center, 2009); Vicki Luker and Jane Buckingham, *Histories of Leprosy: Subjectivities, Community and Pacific Worlds*, special issue of *The Journal of Pacific History* 2017, 265–426.

3 With the exception of South Africa, no serious attempt at introducing compulsory segregation was ever attempted in African colonies: John Iliffe, *The African Poor: A History* (Cambridge: Cambridge University Press, 1987), chapter 12; Megan Vaughan, *Curing Their Ills: Colonial Power and African Illness* (Stanford: Stanford University Press, 1991), esp. chapter 4; Eric Silla, *People Are Not The Same: Leprosy and Identity in Twentieth-Century Mali* (Portsmouth: Heinemann, 1998); David Hardiman, ed., *Healing Bodies, Saving Souls. Medical Missions in Asia and Africa* (Amsterdam: Rodopi, 2006) esp. the chapters by Shankar and Manton; Shobana Shankar, ‘Medical Missionaries and Modernizing Emirs in Colonial Hausaland: Leprosy Control and Native Authority’, *Journal of African History* 2007, 48, 45–68; Kathleen Vongsathorn, ‘Things That Matter’: Missionaries, Government, and Patients in the Shaping of Uganda’s Leprosy Settlements, 1927–1951’ (PhD thesis, University of Oxford, 2012).

4 Cf. ‘Leprosy disappeared, the leper vanished, or almost, from memory; these structures [i.e., exclusion of the leper] remained. Often, in these same places, the formulas of exclusion would be repeated, strangely similar two or three centuries later’: Michel Foucault, *Madness and Civilization: A History of Insanity in the Age of Reason* (New York: Vintage Books, 1988) 57. See also *Ibid*., 202.

5 See Zachary Gussow*, Leprosy, Racism, and Public Health. Social Policy in Chronic Disease Control* (Boulder: Westview Press, 1989) and the discussions in the literature mentioned in notes 2 and 3.

6
*Leprosy, an Imperial Danger*, is the title of an 1889 tract by Henry Press Wright (London: Churchill, 1889).

7 Jo Robertson, ‘In a State of Corruption: Loathsome Disease and the Body Politic’ (PhD thesis, University of Queensland, 1999); Bashford, *Imperial Hygiene*, 81–93; Warwick Anderson, *The Cultivation of Whiteness: Science, Health, and Racial Destiny in Australia* (Durham: Duke University Press, 2006).

8 Edmond, *Leprosy and Empire*.

9 The problem of explaining this difference was discussed in Henk Menke, Stephen Snelders and Toine Pieters, ‘Omgang met lepra in ‘de West’ in de negentiende eeuw. Tegendraadse maar betekenisvolle geluiden vanuit Suriname’, *Studium* 2009, 2, 65–77.

10 Michel Foucault, *The Birth of the Clinic: An Arechaeology of Medical Perception* (London: Tavistock, 1973).

11 Stephen Snelders, *Leprosy and Colonialism: Suriname under Dutch Rule, 1750*–*1950*, Social Histories of Medicine series (Manchester: Manchester University Press, 2017); Leo van Bergen, *Uncertainty, Anxiety, Frugality: Dealing with Leprosy in the Dutch East Indies, 1816*–*1942*, History of Medicine in Southeast Asia series (Singapore: National University of Singapore Press, 2018).

12 On the history of Suriname, see R. A. J. van Lier, *Frontier Society: A Social Analysis of the History of Surinam* (The Hague: Martinus Nijhoff, 1971); Hans Buddingh’, *De geschiedenis van Suriname* (Amsterdam: Nieuw Amsterdam, 2012); Maurits Hassankhan *et al*., eds, *Verkenningen in de historiografie van Suriname*, 2 vols (Paramaribo: Anton de Kom universiteit Suriname, 2013). On plantation colonialism in Suriname, see Martin Schalkwijk, ‘The Plantation Economy and the Capitalist Mode of Production’, in Martin Schalkwijk and Stephen Small, eds, *New Perspectives on Slavery and Colonialism in the Caribbean* (The Hague: Hamrit/Ninsee, 2012), 14–40. For a general overview of Caribbean plantation colonies, see G. Heuman, *The Caribbean* (London: Hodder Arnold, 2006).

13 There were also approximately 500 free ‘colored’ people and mulattos. For the sake of consistency, we will use ‘Europeans’ for all whites, including those born in Suriname (who were called ‘Creoles’ at the time). To avoid confusion, we will use the word ‘Africans’ or ‘Afro-Surinamese’ for slaves and freed people of African descent. Population figures: R. O. Beeldsnijder, ‘Om werk van jullie te hebben’. Plantageslaven in Suriname, 1730–1750 (PhD thesis, Leiden University, 1994), 264–66; B. van der Oudermeulen, ‘Iets tot voordeel der deelgenooten van de Oost-Indische Compagnie en tot nut van ieder ingezetenen van dit gemenebest kan strekken’, in D. van Hogendorp, ed., *Stukken, raakende den tegenwoordige toestand der Bataafsche bezittingen in Oost-Indië en de handel op derzelve* (The Hague: J.C. Leeuwesteyn, 1801), 327–88, 327–28. The year ‘1738’ in the memoir of Van der Oudermeulen is a typo and should be read as ‘1783’.

14 Kenneth F. Kiple, *The Caribbean Slave: A Biological History* (Cambridge: Cambridge University Press, 1984); Richard B. Sheridan, *Doctors and Slaves: A Medical and Demographic History of Slavery in the British West Indies, 1680*–*1834* (Cambridge: Cambridge University Press, 1985). Cf. also Alfred W. Crosby, *The Columbian Exchange: Biological and Cultural Consequences of 1492* (Westport: Greenwood Publishing Co., 1972); Kenneth F. Kiple and Kriemhild Coneé Ornelas, ‘Race, War and Tropical Medicine in the Eighteenth-Century Caribbean’, in David Arnold, ed., *Warm Climates and Western Medicine: The Emergence of Tropical Medicine, 1500*–*1900* (Amsterdam: Rodopi, 1996), 65–79; J. Handler, ‘Diseases and Medical Disabilities of Enslaved Barbadians, from the Seventeenth Century to around 1838’, *The Journal of Caribbean History*, 2006, 37, 1–38, 177–214; J. R. McNeill, *Mosquito Empires: Ecology and War in the Greater Caribbean, 1629*–*1914* (Cambridge: Cambridge University Press, 2010).

15 See M. Worboys, ‘Tropical Diseases’, in W. F. Bynum and Roy Porter, eds, *Companion Encyclopedia of the History of Medicine* (London: Routledge, 1993), vol. 2, 512–36, quotation on 517, on India: ‘In fact, colonialism had little or no knowledge of health and disease amongst indigenous peoples’. On the Dutch East Indies, see Peter Boomgaard, ‘Dutch Medicine in Asia, 1600–1900’, in Arnold, ed., *Warm Climates and Western Medicine*, 42–64.

16 S. Quinian, ‘Colonial Encounters: Colonial Bodies, Hygiene and Abolitionist Policies in Eighteenth-Century France’, *History Workshop Journal*, 1996, 42, 107–26, 112–13.

17 On similar colonial fears of disease returning to the metropole: Alan Bewell, *Romanticism and Colonial Disease* (Baltimore: Johns Hopkins University Press, 1999).

18 On the analytical distinction between ‘center’ and ‘periphery’, see Roy MacLeod, ed., ‘Nature and Empire: Science and the Colonial Enterprise’, *Osiris*, 2000, 15, 1–317. For an example of diffusion from center to periphery, see Sokhieng Au, *Mixed Medicines: Health and Culture in French Colonial Cambodia* (Chicago: The University of Chicago Press, 2011), 157–79.

19 Elizabeth Fox-Genovese and Eugene D. Genovese, *The Mind of the Master Class: History and Faith in the Southern Slaveholders’ Worldview* (Cambridge: Cambridge University Press, 2005), 1.

20 For a fuller analysis, cf. Stephen Snelders, ‘Leprosy and Slavery in Suriname: Godfried Schilling and the Framing of a Racial Pathology in the Eighteenth Century’, *Social History of Medicine*, 2013, 26, 432–50.

21 Boasie is the name of a town in present-day Ghana, about 30 miles to the north of the capital Accra. A Dutch dictionary of 1855 claimed that boasie was the name given in Angola to an ulceration that looked like elephantiasis. Cf. L. C. E. E. Fock, *Natuur- en geneeskundig etymologisch woordenboek* (n.p.: J. Noorduyn, 1855), 170. Other meanings of ‘boasie’ are ‘impure’ and ‘under a rock’: Cf. R. D. G. P. Simons, *Lepra. De lepra-bestrijding in Suriname en de noodzakelijkheid harer reorganisatie* (Amsterdam: Scheltema and Holkema, 1950), 10; R. D. G. P. Simons, *Bijgeloof en lepra in de Atlantische Negerzônes* (Paramaribo: Radhakishun, 1959), 33.

22 Here, ‘leprosy’ is used to refer to ‘boasie’, ‘melaatschheid’ and ‘elephantiasis graecorum’ interchangeably, as these were understood to be synonyms by eighteenth-century physicians, rather than to current medical understandings of Hansen’s disease.

23 On premodern medical thinking with regard to leprosy, see Luke Demaitre, *Leprosy in Premodern Medicine: A Malady of the Whole Body* (Baltimore: The Johns Hopkins University Press, 2007).

24 D. Rolander, ‘Journal’, in L. Hansen *et al*., ed., *The Linnaeus Apostles: Global Science and Adventure*, vol. 3, bd. 3, ed. (London: Ik Foundation, 2008), 1217–564, quotation on p. 1483. Daniel Rolander was a Swedish botanist and entomologist and a student of Linnaeus, who travelled in Suriname in the mid-1750s.

25 ‘Plakkaat, Bescherming tegen besmettelijke ziekten, November 30 1728’, in J. T. de Smidt, ed., *Plakkaten, ordonnantiën en andere wetten, uitgevaardigd in Suriname 1667*–*1816* (Amsterdam: Emmering, 1973), 395–96 and ‘Plakkaat, Voorschriften in verband met besmettelijke ziekten van slaven, February 4 1761’, *ibid*., 707–08.

26 ‘Notifikatie, Het verplegen van slaven in chirurgijnswinkels, February 22 1764’, *ibid*., 780–81.

27 ‘Notifikatie, Zieke of krankzinnige slaven mogen niet worden verkocht, February 10, 1780’, *ibid*., 971–72.

28 ‘Plakkaat, Voorzorgsmaatregelen tegen melaatsheid, May 28/June 4 1790’, *ibid*, 1144–47.

29 Stephen Snelders, *Vrijbuiters van de heelkunde. Op zoek naar medische kennis in de tropen 1600*–*1800* (Amsterdam: Atlas, 2012), 127–29, 156–66.

30 On Schilling, see Snelders, ‘Leprosy and Slavery’. The recent discovery by Natalie Zemon Davis that Schilling was of Dutch origin has to be corrected again. See the updated online version of Natalie Zemon Davis, ‘Physicians, Healers, and Their Remedies in Colonial Suriname’, *Canadian Bulletin of Medical History*, 2016, 33, 3–34, and Natalie Zemon Davis (manuscript in preparation), *Suriname Stories*. The mistake in identifying Schilling with another Schilling living at the same time in Paramaribo was kindly pointed out by Philip Dikland of the Suriname Heritage Guide.

31 G. W. Schilling, *Verhandeling over de melaatsheid* (Utrecht: J.C. ten Bosch, 1771), 4–40.

32
*Ibid*., 29–32. On sexual relations in Suriname between Europeans and Africans, cf. A. A. Stipriaan Luïscius, ‘Surinaams contrast. Roofbouw en overleven in een Caraïbische plantage-economie’ (PhD thesis, Vrije Universiteit Amsterdam, 1991), 396; Buddingh’, *Geschiedenis*, 65–69. Cf. also R. Hyam, *Empire and Sexuality: The British Experience* (Manchester: Manchester University Press, 1990), 92–93; K. K. Weaver, *Medical Revolutionaries: The Enslaved Healers of Eighteenth-Century Saint Domingue* (Urbana: University of Illinois Press, 2006), 18; P. Pluchon, *Nègres et Juifs au XVIIIe siècle. Le racisme au siècle des Lumières* (Paris: Tallandier, 1984), 214.

33 Snelders, ‘Leprosy and Slavery’.

34 M. Foucault, *Madness and Civilization: A History of Insanity in the Age of Reason* (London: Routledge, 2001), 38–40.

35 The analysis in this paragraph is based on Snelders, *Leprosy and Colonialism*, 43–77. The context of segregation in Suriname does not show the ambivalence of for instance early modern Germany: F. Dross and A. Kinzelbach, ‘Nit mehr alls sein burger, sondern alls ein frembdner’. Fremdheit und Aussatz in frühneuzeitlichen Reichsstädten’, *Medizinhistorisches Journal*, 2011, 46, 141–76.

36 A. van Hasselaar, *Beschrijving der in de kolonie* Suriname *voorkomende elephantiasis en lepra (melaatschheid)* (Amsterdam: S. de Greber, 1835), 3–4, 10, 38–41.

37
*Ibid*., 10.

38
*Ibid.*, 73–76.

39 J. Hille, ‘Ueber die *Elephantiasis*; nach eigenen Beobachtungen in West-Indien’, *Wochenschrift für die gesammte Heilkunde* 1841, 433–42, 438.

40
*Gouvernementsblad* 1830, nr. 13.

41
*Gouvernementsblad* 1831, nr. 12.

42
*Surinaamsche almanak voor het jaar 1838* (Paramaribo: Maatschappij tot Nut van ’t Algemeen, 1837), 141.

43
*Gouvernementsblad* 1853 nr. 3; 1855 nr. 8.

44 Source: annual colonial reports of the government to Dutch parliament: *Verslag van het beheer en den staat der Koloniën* (1849–1865); *Koloniaal Verslag* (1866–1897).

45 Cf. Edmond, *Leprosy and Empire*, 44–60. See also Magnus Vollset, ‘Globalizing Leprosy: A Transnational History of Production and Circulation of Medical Knowledge 1850–1930’ (PhD thesis, Bergen University, 2013).

46 For the discussion in the Netherlands, see Snelders, *Leprosy and Colonialism*, 131–35.

47 C. L. Drognat Landré, *De besmettelijkheid der lepra arabum, bewezen door de geschiedenis dezer ziekte in Suriname* (Utrecht: J.L. Beijers, 1867), 72–75.

48
*Ibid.*, 84.

49
*Ibid*., 95.

50 This discussion was reported on in the newspapers in the Dutch Caribbean, e.g. *De Curaçaosche Courant* August 22, 1857.

51
*Staten-Generaal*, Kamerstuk 1861–1862, nr. XXXV, ondernr. 18, www. statengeneraaldigitaal.nl.

52 Van Lier, *Frontier Society*, 190.

53 Rozemarijn Hoefte, *Suriname in the Long Twentieth Century: Domination, Contestation, Globalization* (New York: Palgrave MacMillan, 2014), 1.

54 P. H. J. Lampe and C. Simons, ‘Lepra in Suriname’, *Nederlandsch Tijdschrift voor geneeskunde*, 1929, 73, 4903–15, 4905, 4912.

55 Sources: *Koloniaal Verslag*; *Verslag van bestuur en staat van Suriname* (1924–1930); *Surinaamsch Verslag* (1931–1950); reports Public Health Service: National Archive, Paramaribo, Gouvernementssecretarie Suriname 1829–1954. Inv. nr. 1.01.01. 1258, 1262, 1264, Archive Bisdom of Paramaribo T; G. J. Hallewas, ‘De gezondheidszorg in Suriname’ (PhD thesis, Groningen University, 1981), App. XI (after 1918); Majella 1896–1898: Archive Bisdom of Paramaribo T178.

56 See Snelders, *Leprosy and Colonialism*, 178–85.

57
*Ibid*., 199–218.

58 Femme S. Gaastra, *De geschiedenis van de VOC* (Zuthpen: Walburg Pers, 2002), 64.

59 Peter Harmen van der Brug, ‘Malaria en malaise. De VOC in Batavia in de achttiende eeuw’ (PhD thesis, Leiden University, 1994), 31.

60 van der Brug, *Malaria en malaise*, 33; Peter Boomgaard, ‘Human Capital, Slavery and Low Rates of Economic and Population Growth in Indonesia, 1600–1910’, in Gwyn Campbell, ed., *The Structure of Slavery in Indian Ocean Africa and Asia* (London: Frank Cass, 2004), 83–96; Reggie Baay, *Daar werd wat gruwelijks verricht. Slavernij in Nederlands-Indië* (Amsterdam: Athenaeum – Polak & Van Gennep, 2015), 32–42.

61 Cf. Johan Leonard Blussé van Oud-Alblas, ‘Strange Company: Chinese Settlers, Mestizo Women and the Dutch in VOC Batavia’ (PhD thesis, Leiden University, 1986). On the population of Batavia also: van der Brug, *Malaria en malaise*, 31–36; Jean Gelman Taylor, *The Social World of Batavia: European and Eurasian in Dutch Asia* (Madison: The University of Wisconsin Press, 1983).

62 van der Brug, *Malaria en malaise*, 36.

63 J. J. P. de Jong, *De waaier van het fortuin. Van handelscompagnie tot koloniaal imperium* (The Hague: Sdu, 1998) 125, 129–31, 140.

64 Wilhem ten Rhyne, *Verhandelinge van de Asiatise melaatsheid na een naukeuriger ondersoek, ten dienste van het gemeen* (Amsterdam: Abraham van Someren, 1687), 70.

65 ‘Instructie voor de buiten-regenten van het Lazarus-huis, August 5, 1667’, in J. A. van der Chijs, ed., *Nederlandsch-Indisch Plakaatboek*, *1642*–*1677*, (Batavia: Landsdrukkerij, 1886), vol. 2, 430–33.

66
*Ibid*., *1602*–1811, vol. 3, 73.

67 J. M. H. van Dorssen, ‘De lepra in Nederlandsch Oost-Indië tijdens de 17^e^ en 18^e^ eeuw’, *Geneeskundig Tijdschrift voor Nederlandsch-Indië* 1897, 37, 255–324, 284–87.

68 ‘Voorschriften nopens emancipatie van lijfeigenen bij acte onder levenden en nopens personen, besmet met “de sieckte van lazarye”, January 15, 1682’, point 4, in Van der Chijs, ed., *Nederlandsch-Indisch Plakaatboek*, vol. 3, 75–79, 78–79.

69 J. M. H. van Dorssen, ‘Dr. Wilhelm ten Rhijne and leprosy in Batavia in the 17th century’, *Janus*, 1898, 2, 252–60, 256–58.

70 Ten Rhyne, *Verhandelinge*; van Dorssen, ‘Dr. Wilhelm ten Rhijne’; Harold J. Cook, *Matters of Exchange: Commerce, Medicine, and Science in the Dutch Golden Age* (New Haven: Yale University Press, 2007), 361–62.

71 Ten Rhyne, *Verhandelinge*, 98.

72
*Ibid*., 94.

73 van Dorssen, ‘De lepra’, 293.

74 In 1690, there were 171 patients on Purmerend (of whom 95 were slaves, 69 natives and 7 Europeans). In 1713, there were 109 (75 slaves, 23 natives and 5 Europeans) and in 1764 there were 51 (29 slaves, 12 natives, 5 Chinese and 2 Europeans): *Ibid*., 314.

75 The asylum was formally closed in 1803: *Ibid*., 301–05.

76 Overviews of the history of the Dutch East Indies include H. W. Van den Doel, *Het Rijk van Insulinde. Opkomst en ondergang van een Nederlandse kolonie* (Amsterdam: Prometheus, 1996); de Jong, *Waaier van het fortuin*; J. A. A. van Doorn, *De laatste eeuw van Indië* (Amsterdam: Bert Bakker, 1994).

77 A. H. M. Kerkhoff, ‘De organisatie van de militaire en burgerlijke geneeskundige dienst in de 19de eeuw’, in G. M. van Heteren, A. de Knecht-van Eekelen and M. J. D. Poulissen, eds, *Nederlandse geneeskunde in de Indische archipel 1816*–*1942* (Amsterdam: Rodopi, 1989), 7–22; Susan Abeyasekere, ‘Death and Disease in Nineteenth Century Batavia’, in Norman G. Owen, ed., *Death and Disease in Southeast Asia: Explorations in Social, Medical and Demographic History* (Singapore: Oxford University Press, 1987), 189–210, 197; Liesbeth Hesselink, *Healers on the Colonial Market: Native Doctors and Midwives in the Dutch East Indies* (Leiden: KITLV Press, 2011).

78 Abeyasekere, *ibid*., 196, 206.

79 Hans Pols, ‘Quarantine in the Dutch East Indies’, in Alison Bashford, ed., *Quarantine: Local and Global Histories*, (Basingstoke: Palgrave Macmillan, 2016), 85–102.

80 D. Schoute, ‘De geneeskunde in Nederlandsch-Indië gedurende de negentiende eeuw’, part 6, ‘De geneeskunde tusschen 1823 en 1845 residentie-gewijze beschouwd’, *Geneeskundig Tijdschrift voor Nederlandsch-Indië* 1934, 74, 1720–35, 1732; D. Schoute, *De geneeskunde in Nederlandsch-Indië gedurende de negentiende eeuw* (Batavia: G. Kolff, 1934), 328; D. Schoute, ‘Enkele volksplagen in het verleden van Nederlandsch Indië’, part 4, ‘Lepra’, *Nederlandsch Tijdschrift voor Geneeskunde* 1935, 79, 1587–604, 1594–97; D. Schoute, *Occidental Therapeutics in the Netherlands East Indies During Three Centuries of Netherlands Settlement (1600*–*1900)* (Batavia: Netherlands Indian Public Health Service, 1937), 174.

81 Only in the few territories which the Dutch controlled leprosy asylums were founded: apart from Java, this included Sumatra, the Moluccas and Sulawesi: van Bergen, *Uncertainty*, 40–60.

82 ‘Resident’ is a title referring to a Dutch administrator who was the highest local representative of the Governor General. In total, Java had some 20 Residencies.

83 Report G. Wassink, Head of the health service, 1–9, in Archive D. Schoute, Inst. A5, Map IV, Boerhaavemuseum, Leiden; Schoute, ‘Enkele volksplagen’, 1600–1.

84 Snelders, *Leprosy and Colonialism*, 213–14.

85
*Gouvernements Besluit* September 29, 1865, Nr. 11. Cf. also *Verslag van het beheer en den staat van Nederlandsch Indië over 1865*, Kamerstuk Tweede Kamer der Staten-Generaal nr. 58, ondernr. 2, 683 (www.statengeneraaldigitaal.nl); E. W. A. Ludeking, *Recueil voor den civiel geneeskundigen dienst in Nederlandsch-Indië* (Batavia: Van Dorp, 1871), 96.

86
*Gouvernements Besluit* September 15, 1870, Nr. 59; Ludeking, *ibid.*, 97.

87 van Bergen, *Uncertainty*, 66–74, 132.

88 B. M. van Driel, ‘Over behandeling en bestrijding van lepra’, *Geneeskundig Tijdschrift voor Nederlandsch-Indië*, 1922, 62, 149–63, 159–60. See also *Handelingen Staten-Generaal*, Kamerstuk Tweede Kamer 1905–1906, kamerstuknummer 5, number 1: Koloniaal Verslag van 1905, Nederlandsch-Indië, Appendix P, 10, www.statengeneraaldigitaal.nl.

89
*Mittheilungen und Verhandlungen der internationalen wissenschaftlichen Lepra-Conferenz zu Berlin*, 3 vols. (Berlin: August Hirschwald, 1897); S. Pandya, ‘The First International Leprosy Conference, Berlin 1897’, *Manguinhos*, 2003, 10, 161–77; Edmond, *Leprosy and Empire*, 103–07; Vollset, ‘Globalizing Leprosy’. For the role of doctors from the Dutch East Indies, cf. van Bergen, *Uncertainty*, 96–103.

90 C. L. van der Burg, *De geneesheer in Nederlandsch-Indië*, vol. 2, *Pathologie en therapie der ziekten in Nederlandsch-Indië* (Batavia: Ernst, 1887), 430.

91 ‘Lepra-lijders in Indië’, *De Indische Gids*, 1894, 16, 140–41; Van Bergen, *Uncertainty*, 116–17; H. van Kol. ‘Het Lepra-gevaar’, *De Indische Gids*, 1898, 20, 1249–67, 1262.

92 For example, van Kol, ‘Lepragevaar’, 1251–52; ‘Lepra hier en in de Oost’, *Algemeen Handelsblad*, 2 November 1898, evening edition; ‘Land- en Mijnbouw’, *Het Nieuws van den Dag voor Nederlandsch-Indië*, 20 October 1900, complained about the presence of a Chinese leprosy sufferer on a tram. In ‘Op den uitkijk’, *De Sumatra Post*, 8 August 1903, a colonist in Medan expressed fears of becoming completely dependent for his vegetables on his Chinese greengrocer who suffered from leprosy; medical view: R. Römer, ‘Melaatschheid en hare bestrijding door afzondering’, *Medisch weekblad*, 1916, 23, 337–40, 349–52, 361–66, 340.

93 Cf. ‘The West changed the traditional Chinese self-image by imposing the interpretation that … China was at the margin of the Western civilized world. The Chinese had to face the fact that they were now the inferior, Oriental, “other”’: Leung, *Leprosy in China*, 215. See also Gussow, *Leprosy, Racism, and Public Health*.

94 ‘De lepra en de school’, *De Sumatra post*, 25 May 1908. *Staatsblad voor Nederlandsch-Indië*, Bijblad nr. 6758, 23 October 1907, 19: ‘Besluit Lepralijders’, *Staatsblad voor Nederlandsch-Indië*, Bijblad 6853, 7 April, 1908, 185–86.

95 van den Doel, *Rijk van Insulinde*, 115–24; Jan Breman, *Koelies, planters en koloniale politiek* (Leiden: KITLV Uitgeverij, 1992); Ann Laura Stoler, *Capitalism and Confrontation in Sumatra’s Plantation Belt* (New Haven: Yale University Press, 1985).

96 Stoler, *Capitalism and Confrontation*, 28. On the coolie ordinance cf. also Baay, *Daar werd wat gruwelijks verricht*, 239–45.

97 Cf. van Doorn, *Laatste eeuw*, 25–26.

98 ‘Een-en-ander. Al bladerende…’, *De Sumatra post*, 21 February 1910, Morning edition; ‘Vreemde smitten’, *De Sumatra post*, 25 October 1910, writes of ‘Chinese vampires’.

99 Cf. van Doorn, *Laatste eeuw*, 91–97.

100 J. A. Voorthuis, ‘Verslag van de Wetenschappelijke werkzaamheden van de onderafdeeling Sumatra’s oostkust der vereeniging tot bevordering van geneeskundige wetenschappen’, *Geneeskundig Tijdschrift voor Nederlandsch-Indië*, 1897, 37, 325–30, 326.

101 R. Römer, ‘La lepre’, *Janus*, 1904, 9, 567–74, 627–34, 1905, 10, 37–42, 79–87, 150–6, 205–15, 208–10.

102 Anonymous, ‘Eenige statistische en andere gegevens, ontleend aan de civiel geneeskundige jaarverslagen en aan de rapporten omtrent besmettelijke ziekten in Nederlandsch-Indiö over het jaar 1906. Tweede deel’.

103 Römer, ‘Melaatschheid en hare bestrijding’, 350.

104
*Staatsblad* 1913 nr. 44 art. 230: Archive of the National Republic of Indonesia (ANRI), Jakarta: Algemene secretarie, CI 1-8-1912, nr. 18446; MGS 16-101912, nr. 287. Cf. also H. G. van Eelen, ‘Het lepra-vraagstuk’, *Nieuwe Rotterdamsche Courant*, 7 December 1926, evening edition.

105 ‘Verslag van het wetenschappelijk gedeelte der vergaderingen van de afdeling Sumatra’s oostkust’, *Geneeskundig Tijdschrift voor Nederlandsch-Indië*, 1917, 57, xxix–xxx, xxx.

106 van Bergen, *Uncertainty*, 165–66.

107 J. J. van Lonkhuyzen, ‘Het lepravraagstuk’, *Geneeskundig Tijdschrift voor Nederlandsch-Indië*, 1937, 77, 834–58, 838.

## Notes

Stephen Snelders is a Dutch historian and Research Fellow at University Utrecht, Faculty of Science, Freudenthal Institute/History and Philosophy of the Sciences, The Netherlands. He has published numerous books and articles on the history of drugs and addiction, on tropical medicine and piracy and on human genetics and eugenics. His most recent book is *Leprosy and Colonialism: Suriname under Dutch Rule, 1750*–*1950* (Manchester University Press, 2017), which deals with tropical medicine and race in a colonial setting.

Leo van Bergen is an independent Dutch medical historian with a special interest in war and medicine, and tropical medicine. Among his several books are a medical history of the First World War (*Before my Helpless Sight*), a work on Red Cross aid in the Dutch East Indies (*On Humanitarianism and Colonialism*) and a book on leprosy in that same former Dutch colony (*Uncertainty, Anxiety, Frugality*).

Frank Huisman is a Professor in the History of Medicine at the University Medical Center of Utrecht University and also Chair of the Scientific Board of the European Association for the History of Medicine and Health. He has published on early modern and modern Dutch medicine and health care and on medical historiography. He is now working on a book exploring the transformation of Dutch health care between 1865 and 1941. Recently, he co-edited *Blurring Boundaries: Towards a Medical History of the Twentieth Century* and a Handbook on medical history (in Dutch). Huisman was the supervisor of the NWO-funded comparative research project ‘Leprosy and Empire’. Recently, he set up a global research network working on ‘Governance and Accountability in Global Health, post World War II’.

